# Development and Surface Indentation Assessment of an MRI-Based Subject-Specific Hand–Forearm Finite Element Model: A Preliminary Study

**DOI:** 10.3390/bioengineering13091015

**Published:** 2026-08-31

**Authors:** Songhao Chen, Lei Jia, Shuang Ren, Changqing Ke, Qiguo Rong

**Affiliations:** 1 School of Mechanics and Engineering Science, Peking University, Beijing 100871, China; lijfuj@stu.pku.edu.cn; 2 Beijing Xiaomi Mobile Software Co., Ltd., Xi’erqi Middle Road, Haidian District, Beijing 100085, China; jialeijlu@163.com (L.J.); kechangqing@xiaomi.com (C.K.); 3 Department of Sports Medicine, Peking University Third Hospital, Institute of Sports Medicine of Peking University, Beijing 100191, China; xixishuang123@126.com; 4 Beijing Key Laboratory of Research and Translation for Drugs and Medical Devices in Precision Diagnosis and Treatment of Sports Injuries, Beijing 100191, China; 5 Engineering Research Center of Sports Trauma Treatment Technology and Devices, Ministry of Education, Beijing 100191, China

**Keywords:** hand and forearm, MRI, finite element model, surface indentation, layered soft tissue

## Abstract

Background: Excessive or poorly distributed contact loading of the hand and forearm can cause pain, functional limitation, skin breakdown, and deep soft-tissue injury, motivating reliable quantification of local soft-tissue deformation and stress. Although anatomically detailed finite element models of the hand and forearm have been developed separately, their integration within a continuous subject-specific hand–forearm model remains limited. Methods: This preliminary single-subject study developed an MRI-based subject-specific finite element model of the hand and forearm from one healthy adult male volunteer. The model incorporated the complete anatomical structures of the hand and forearm, together with the humerus, which provided proximal attachment sites for the forearm muscles and the extrinsic muscles of the hand. Selected muscle–tendon paths were refined using published hand–wrist musculoskeletal parameters. Skin-, fat-, and muscle-thickness fields were quantified using an anatomy-informed thickness-estimation framework centered on a modified bidirectional ray-projection (mBRP) procedure. Representative indentation sites were selected by thickness fields. A material combination selected from the P1 and P2 analyses was evaluated unchanged at the held-out P3 site, followed by exploratory post hoc screening at P3; agreement was assessed using root-mean-square error (RMSE), normalized root-mean-square error (NRMSE), coefficient of determination (R^2^), and relative absolute peak-force error. Results: The model comprised 179 components, including bones, muscles, ligaments, tendons, cartilage, skin, and subcutaneous fat, with 120,563 tetrahedral elements and 56,944 pentahedral wedge elements. All five anthropometric measurements differed by less than 5% from the male P50 reference values. In total, 27 of the 29 comparable muscle-belly lengths fell within published ranges or agreed with available single-specimen values. The mBRP-derived skin-thickness measurements differed from the prescribed values by only 0.00–1.67%. The best-fitting combinations were Skin2–Fat2 at P1 ($R^{2}$ = 0.972) and Skin3–Fat3–Muscle3 at P2 ($R^{2}$ = 0.973). The Skin3–Fat2–Muscle3 combination retained good agreement at the held-out P3 site ($R^{2}$ = 0.877). In comparison, the P2-optimal Skin3–Fat3–Muscle3 combination showed substantially poorer agreement at P3 (R^2^ = 0.344). Subsequent exploratory screening of all 27 combinations at P3 identified Skin2–Fat2–Muscle3 as the closest site-specific fit (R^2^ = 0.983). Conclusions: Within this preliminary single-subject study, the model showed anatomical consistency and reproduced the passive indentation responses at the three investigated sites. Cross-site transferability was material-combination dependent: the jointly selected P1–P2 combination retained good agreement at P3, whereas the P2-specific optimum did not. This study demonstrates the technical feasibility of an individualized hand–forearm modeling workflow but does not establish universally transferable tissue parameters or population-level generalizability. Evaluation in additional participants is required.

## 1. Introduction

The hand and forearm are fundamental to fine motor control, functional grasping, and activities of daily living. Early finite element studies in hand and wrist orthopedics primarily focused on osseous structures [1,2,3]. However, osseous-only representations are insufficient when the modeling objective requires consideration of the mechanical contributions of muscles, joints, and surrounding soft tissues [4,5]. Accordingly, upper-limb biomechanical modeling requires a transition from isolated bone-based representations toward integrated musculoskeletal system models.

Finite element models of the upper extremity have been applied across diverse biomechanical problems [6,7,8,9,10]. Nevertheless, many existing models remain limited to the fingertip, a specific grasping task, or a single contact region, and therefore do not capture the continuous muscle–tendon pathways extending from the forearm muscle bellies through the wrist and palm to the finger attachments. This limitation constrains their applicability to clinical and preclinical scenarios in which contact loading, internal tissue deformation, and musculoskeletal support interact across the hand–forearm complex.

A further challenge is that clinically relevant contact mechanics are not governed by the skin surface alone. External contact loads are transmitted through the skin, subcutaneous fat, muscle, tendons, and underlying bone. Experimental–MRI and finite element studies of pressure-related deep-tissue injury have shown that external contact can generate internal mechanical conditions that are not adequately represented by surface pressure alone [11,12]. In a review of finite element modeling studies of finger and hand mechanical behavior during haptic interactions, Cei et al. emphasized that soft-tissue geometry, material properties, and contact boundary conditions are key determinants of simulated hand contact responses [13]. Recent high-fidelity forearm musculoskeletal modeling has similarly demonstrated the biomechanical value of detailed anatomical representation [14]. Thus, for preclinical contact assessment, hand–forearm models should incorporate layered soft tissues and deep anatomical support rather than treating the limb as a simplified surface envelope.

Surface indentation offers a practical experimental approach for evaluating passive soft-tissue compression. Lu and Zheng used a two-dimensional finite element model to analyze soft-tissue indentation over a curved rigid substrate and showed that substrate curvature, indentation depth, and the ratio of indenter diameter to tissue thickness can affect the accuracy of Young’s modulus estimation from indentation testing [15]. Iivarinen et al. combined in vivo forearm indentation experiments with finite element analysis and demonstrated that indenter geometry, skin thickness, and the mechanical properties of different soft-tissue layers influence the measured response of a manual indentation device and its sensitivity to deep-tissue stiffness changes [16]. Neumann et al. used instrumented ultrasound indentation data from 95 participants across eight upper- and lower-extremity sites and reported substantial regional variation in the in vivo surface stiffness of extremity soft tissues [17]. Their subsequent work further showed that multilayer tissue composition and layer thickness, including those of skin, fat, and muscle, influence predictions of surface indentation responses and layer deformation in musculoskeletal extremities [18]. Force–displacement curves therefore should not be interpreted as direct measurements of the material properties of a single tissue layer, but rather as integrated responses reflecting tissue material behavior, local layer thickness, surface geometry, and deep boundary conditions.

Previous studies have separately developed subject-specific hand models, high-fidelity forearm models, and MRI-based layered indentation models. Wei et al. developed a subject-specific muscle-driven hand model [9], Shang et al. constructed a detailed forearm musculoskeletal model [14], Sengeh et al. combined MRI-derived layered geometry with in vivo indentation and finite element analysis [19], and Wu et al. investigated layered fingertip compression [20]. These representative models are compared in Supplementary Section S1. The principal contribution of the present study is the development of an anatomically detailed subject-specific model integrating the hand and forearm, including layered soft tissues and continuous forearm-to-hand muscle–tendon pathways. The indentation experiments were used to evaluate the force–displacement response of the developed model.

This preliminary single-subject study aimed to develop an MRI-based subject-specific finite element model of the hand and forearm that integrates continuous anatomy, layered soft tissues, and muscle–tendon geometry, and to evaluate response-level agreement between its predicted passive indentation responses and the corresponding in vivo measurements in the investigated participant. Representative surface regions with distinct layer-thickness characteristics were selected to screen literature-derived material combinations at P1 and P2 and to assess the combination-dependent cross-site performance of the selected candidate at P3 without further parameter adjustment.

## 2. Materials and Methods

### 2.1. Participant and MRI Data

Model construction was based on anthropometric data from an adult male whose body dimensions closely matched the male P50 values reported in GB/T 10000-2023 [21], the Chinese National Standard for Human Dimensions. This study was approved by the Ethics Committee of Peking University Third Hospital (No. M2024688), and written informed consent was obtained before data collection. Five structural measurements, namely hand length, hand breadth, elbow–wrist length, index-finger length, and proximal index-finger breadth, were used as reference indicators for geometric alignment.

Relative deviation was calculated as

(1) $$\delta =\left(x_{\mathrm{study}}-x_{P50}\right)/x_{P50}\times 100\%.$$

The geometry was considered representative of the P50 reference when all five indicators were within ±5% of the corresponding standard values.

MRI data were acquired using a 3.0-T United Imaging uMR 880 scanner (Shanghai United Imaging Healthcare Co., Ltd., Shanghai, China). To balance anatomical coverage with local spatial resolution, three scans were obtained: a high-resolution hand scan, a high-resolution forearm scan, and a low-resolution whole hand–forearm scan for registration. The hand scan had a field of view of 159.9 mm, an in-plane pixel size of 0.542 mm, and a slice thickness of 0.81 mm. The forearm scan had a field of view of 160.0 mm, an in-plane pixel size of 0.667 mm, and a slice thickness of 0.81 mm. The whole hand–forearm scan had a field of view of 159.7 mm, an in-plane pixel size of 0.726 mm, and a slice thickness of 1.0 mm.

### 2.2. Anatomical Reconstruction

Model geometry was initially segmented from the MRI data in Mimics and subsequently refined in Blender to correct local anatomical discontinuities, tendon paths, and muscle trajectories. Because of the complex morphology of the intrinsic and extrinsic muscle groups and the limited visibility of several tendon paths on MRI slices, muscle–tendon trajectories were manually adjusted in Blender according to published attachment sites.

Established upper-extremity musculoskeletal models provide a reference framework for representing multi-joint muscle–tendon pathways [22]. For the present hand–wrist geometry, the anatomical dataset reported by Mirakhorlo et al. was specifically used to guide path correction for selected intrinsic and extrinsic muscle–tendon units (Table 1) [23].

The final reconstructed anatomy included bones, muscles, ligaments, cartilage, tendons, subcutaneous fat, and skin. The FPB and APB could not be clearly distinguished on MRI; therefore, because of their similar anatomical origins, they were represented as a single composite muscle unit in the model. For each muscle–tendon unit, the model recorded the origin, insertion, path control points, muscle-belly length, tendon length, and total muscle–tendon path length. These geometric variables characterize the anatomical completeness of the model.

### 2.3. Layered Soft-Tissue-Thickness Fields

**Figure 1:**
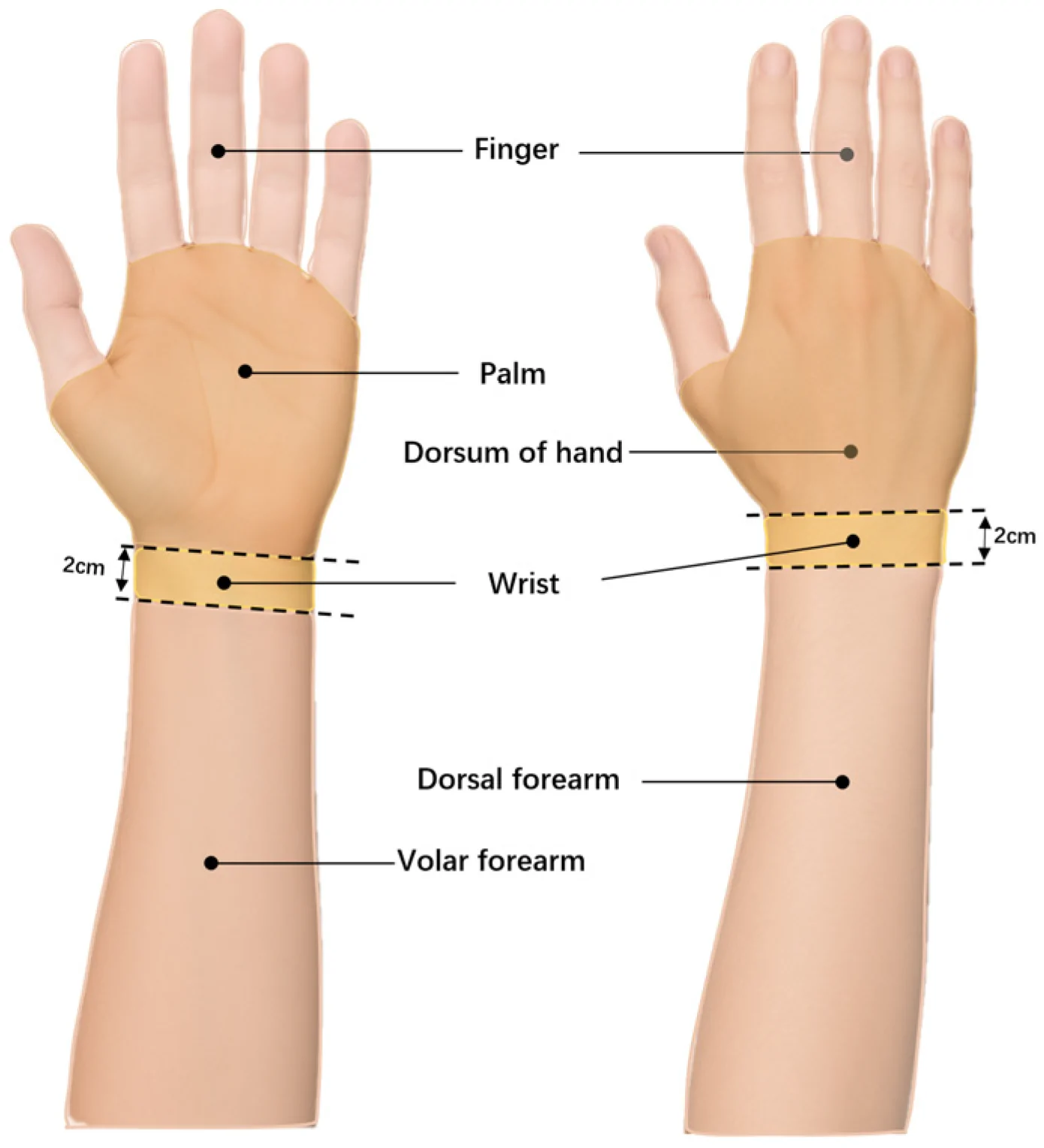
Skin-region partitioning and surface-region definition.

The skin was partitioned into anatomical regions, including the volar forearm, dorsal forearm, wrist, dorsum of the hand, palm, and fingers (Figure 1). The skin layer was generated through geometric offsetting (Table 2), whereas the spatial-thickness distributions of fat and muscle were calculated from the reconstructed anatomical geometries.

Ray-projection approaches have been used to estimate tissue or geometric thickness [27]. For the highly curved and locally concave geometry of the hand and forearm, a modified bidirectional ray-projection (mBRP) procedure incorporating a three-stage screening strategy was implemented. A detailed description of the procedure is provided in Supplementary Section S2.

The regional thicknesses of the skin layer were prescribed a priori during geometric reconstruction and therefore provided known reference values for evaluating the accuracy of the mBRP in measuring soft-tissue thickness. As shown in Table 3, the thicknesses calculated using this method differed from the prescribed values by only 0.00–1.67%. Following this verification, the mBRP was applied to calculate the thickness distributions of the fat and muscle layers, whose thicknesses had not been prescribed in advance.

Following verification, the mBRP was applied to calculate the fat- and muscle-thickness fields. The prescribed skin thicknesses and the resulting fat- and muscle-thickness fields were then used to select three indentation sites: a skin-dominant site (P1), a high-fat-thickness site (P2), and a high-muscle-thickness site (P3).

The selection of P1 required additional consideration because the palm had the greatest prescribed full-thickness skin value. However, the MRI data were acquired with the participant in a prone position and the palm facing downward. Contact with the supporting surface produced visible palmar compression, which could have altered the local geometry and tissue thickness. Therefore, P1 was defined as a skin-dominant site rather than the globally thickest-skin site. Within the remaining non-compressed regions, a skin-dominance index was calculated as the ratio of the prescribed skin thickness to the total local soft-tissue thickness:

$$D_{\mathrm{skin}}=\frac{t_{\mathrm{skin}}}{t_{\mathrm{skin}}+t_{\mathrm{fat}}+t_{\mathrm{muscle}}}$$

where $D_{\mathrm{skin}}$ is the skin-dominance index, and $t_{\mathrm{skin}}$, $t_{\mathrm{fat}}$, and $t_{\mathrm{muscle}}$ are the local thicknesses of the skin, fat, and muscle layers, respectively.

### 2.4. Meshing and Material Properties

Cartilage tissues were discretized using pentahedral elements, whereas all remaining anatomical structures were discretized using tetrahedral elements.

Elastic parameters for bone and cartilage were assigned according to previous mechanical characterizations [28,29]. Ligament behavior was defined using neo-Hookean parameters derived from ligament testing [30].

All soft tissues were assumed to undergo large deformation and to be nearly incompressible. The skin was modeled as a single homogeneous layer, without separate representation of the epidermis and dermis. For the three volume-dominant tissue categories, namely skin, fat, and muscle, three candidate hyperelastic constitutive models were selected from the literature for each category. The strain energy functions and corresponding literature-based parameter ranges for each candidate model are summarized in Table 4.

Because complete constitutive datasets for hand and forearm tissues under matching indentation conditions were unavailable, the candidate parameter library included sources that differed from the present study in anatomical site, species, or loading regime. Each candidate was implemented as a complete literature-derived constitutive configuration, comprising its constitutive form, parameter values, and, where applicable, viscoelastic terms. Therefore, the screening compared the overall predictive performance of complete configurations and was not intended to separate the individual effects of constitutive form, parameter magnitude, or rate dependence.

The indentation experiment was designed to characterize the passive mechanical response of the multilayer soft tissues under local compression. Because no voluntary muscle contraction or joint movement was involved, active muscle activation and activation-dependent contractile stress were not prescribed in the finite element simulations.

### 2.5. Surface Indentation Experiment

Three representative indentation points were selected from the thickness fields. In vivo indentation is an established method for characterizing the mechanical response of soft tissues under controlled compression [39]. Testing was performed using an electronic push–pull force gauge (Z series, AIGU, Hong Kong, China) and a custom-made, 3D-printed cylindrical indenter. The indenter had a diameter of 26 mm, a thickness of 1.1 mm, and a 0.5 mm edge fillet. The force gauge and indenter were coaxially mounted in a vertical guiding device to align the indentation direction as closely as possible with the local surface normal. Each site was marked on the skin surface, and displacement-controlled indentation was applied at a nominal rate of 0.5 mm/s. The total prescribed indenter displacements were 2, 5, and 3.5 mm at P1, P2, and P3, respectively. After subtracting the pre-contact displacement required to close the initial gaps of 0.4, 0.5, and 0.3 mm, the corresponding net indentation depths were 1.6, 4.5, and 3.2 mm.

The force gauge was zeroed and calibrated before testing. During indentation, the participant was seated with the tested upper limb resting naturally on a horizontal support platform. The forearm and hand were kept relaxed, and the participant maintained the prescribed position voluntarily without additional external fixation. A 60 s rest interval was provided between repeated measurements.

**Figure 2:**
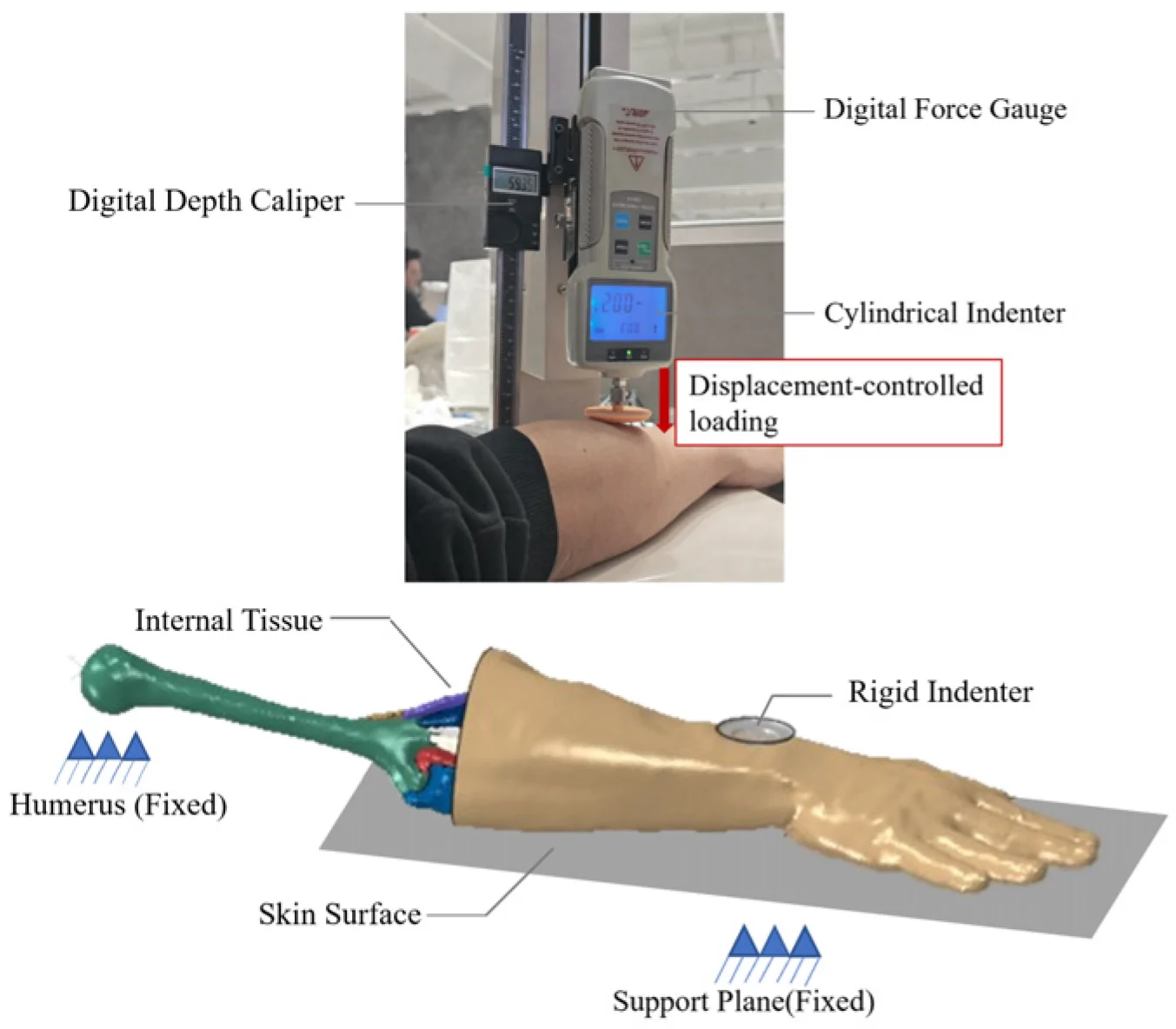
In vivo indentation apparatus and finite element indentation boundary conditions.

Each point was measured three times, and the mean force–displacement curve was used as the experimental reference for model–experiment comparison (Figure 2). The corresponding experimental peak force at the maximum net indentation was reported as the mean ± SD across the three repeated measurements.

### 2.6. Boundary Conditions and Finite Element Simulation

To connect the different model components, shared-node coupling was applied at all bone–cartilage interfaces, ligament–bone insertions, tendon–bone insertions, and skin–fat interfaces. Sliding contact was defined between different anatomical components and within individual structures.

Finite element analyses were performed using the implicit solver in Abaqus (Dassault Systèmes SIMULIA Corp., Johnston, RI, USA). A geometrically nonlinear Static, General step was employed with NLGEOM enabled. The step time was set equal to the total analysis duration, and the simulations were treated as quasi-static analyses. Automatic time incrementation was used. Simulations used the same indenter geometry and displacement boundary conditions as the in vivo experiment (Figure 2). The indenter and the support plane were modeled as rigid. The support plane, model base, and proximal humerus were fully constrained. The indenter was allowed to move only along the local indentation direction, while its remaining degrees of freedom were constrained. Indentation was applied using a smooth-step displacement amplitude, and the corresponding reaction force was extracted from the RF3 history output at the indenter reference point.

### 2.7. Quantitative Agreement Metrics and Cross-Site Evaluation

Model performance was evaluated by comparing experimentally measured and numerically simulated force–displacement (F–u) curves at the selected indentation locations. For P1 and P2, these comparisons were used for material-set selection. At P3, the comparison of the experimental and simulated F–u curves obtained using the P1–P2-selected material set to assess its cross-site predictive transfer-ability.

Following this held-out assessment, additional material sets were evaluated at P3 as a post hoc exploratory analysis.

To enable point-wise comparison, total indenter travel was converted to net indentation by subtracting the pre-contact gap. Experimental and simulated curves were linearly interpolated onto a common displacement grid with an interval of 0.01 mm. The site-specific comparison ranges, $\left[0,u_{max}\right]$, were [0, 1.6] mm for P1, [0, 4.5] mm for P2, and [0, 3.2] mm for P3. $F_{exp}\left(u_{i}\right)$ denotes the mean experimental force across the three repetitions at displacement $u_{i}$, whereas $F_{\mathrm{sim}}\left(u_{i}\right)$ denotes the corresponding simulated force. Agreement between $F_{exp}(u)$ and $F_{\mathrm{sim}}(u)$ was then quantified using four complementary metrics: (i) the root-mean-square error (RMSE),

(2) $$RMSE=\sqrt{\frac{1}{n}\sum_{i=1}^{n}\;{\left(F_{exp}\left(u_{i}\right)-F_{\mathrm{sim}}\left(u_{i}\right)\right)}^{2}}$$

and (ii) normalized RMSE (NRMSE),

(3) $$\mathrm{NRMSE}=\mathrm{RMSE}/\left(F_{\exp,\;max}-F_{\exp,\;min}\right)$$

to enable comparison across sites with different force magnitudes; (iii) the coefficient of determination,

(4) $$R^{2}=1-\frac{\sum_{i}\;{\left(F_{exp}-F_{sim}\right)}^{2}}{\sum_{i}\;{\left(F_{exp}-{\overset{‾}{F}}_{exp}\right)}^{2}}$$

to assess agreement in curve shape; and (iv) the relative absolute peak-force error at the maximum indentation depth (hereafter referred to as the peak-force error),

(5) $$\varepsilon_{F,max}=\frac{\left|F_{sim}\left(u_{max}\right)-F_{exp}\left(u_{max}\right)\right|}{F_{exp}\left(u_{max}\right)}$$

The transfer candidate was selected exclusively from the P1 and P2 screening results, without reference to the P3 results. NRMSE and $R^{2}$ were used as the primary selection criteria, whereas RMSE and peak-force error were used as supporting indicators.

## 3. Results

### 3.1. Participant Measurements

The participant’s measured dimensions showed close agreement with the male P50 reference values reported in GB/T 10000-2023 (Table 5). Deviations for all five anthropometric indicators remained within ±5%, supporting the suitability of this participant for constructing an approximately average-sized adult male hand–forearm model.

### 3.2. Anatomical Composition and Mesh Characteristics

**Figure 3:**
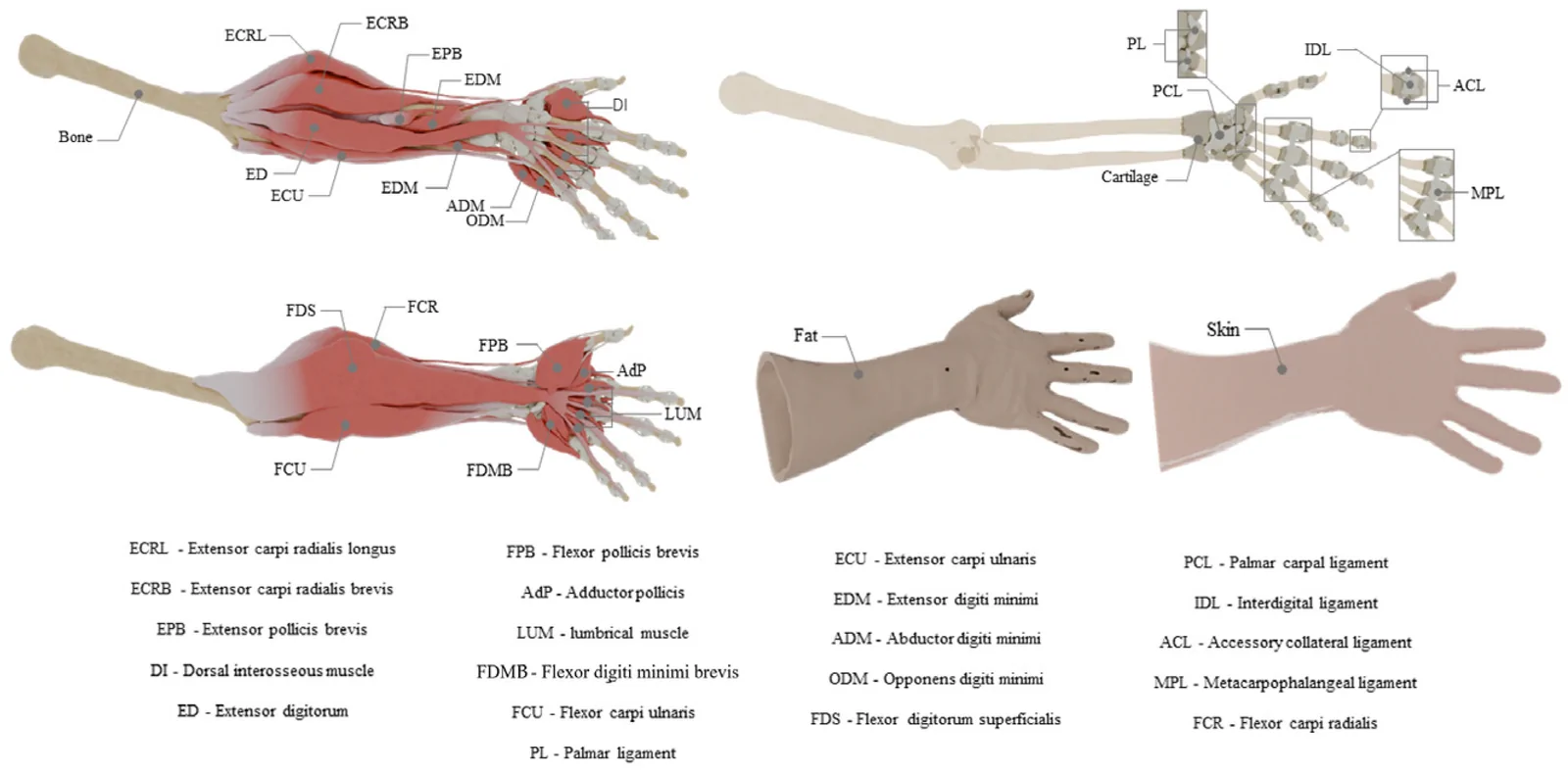
Anatomical composition of the MRI-based hand–forearm finite element model, including skin, subcutaneous fat, bone, muscle, tendon, and ligament structures.

The hand–forearm finite element model represented the major anatomical structures extending from the hand to the proximal forearm. The model comprised 179 components, including 30 bones, 30 muscles, 49 ligaments, tendons, cartilage, skin, and subcutaneous fat (Figure 3). The osseous structures included the carpal bones, metacarpals, phalanges, and arm bony supports; the muscular structures included both extrinsic forearm muscles and intrinsic hand muscles; and the ligament structures represented the major periarticular constraints.

Because FPB and APB were modeled as one composite unit, the 30 anatomical muscles corresponded to 29 comparable muscle-belly units. Of these, 27 were within reported ranges or consistent with available single-specimen values. AbPL (126.87 mm versus 134–176 mm) and PI 2 (55.35 mm versus 45.3–51.1 mm) were the two exceptions (Table 6).

The mesh included 120,563 tetrahedral elements and 56,944 pentahedral wedge elements. Details of the local mesh-convergence assessment at P1, P2, and P3 are provided in Supplementary Section S3.

### 3.3. Layered Soft-Tissue-Thickness Fields and Representative Points

**Figure 4:**
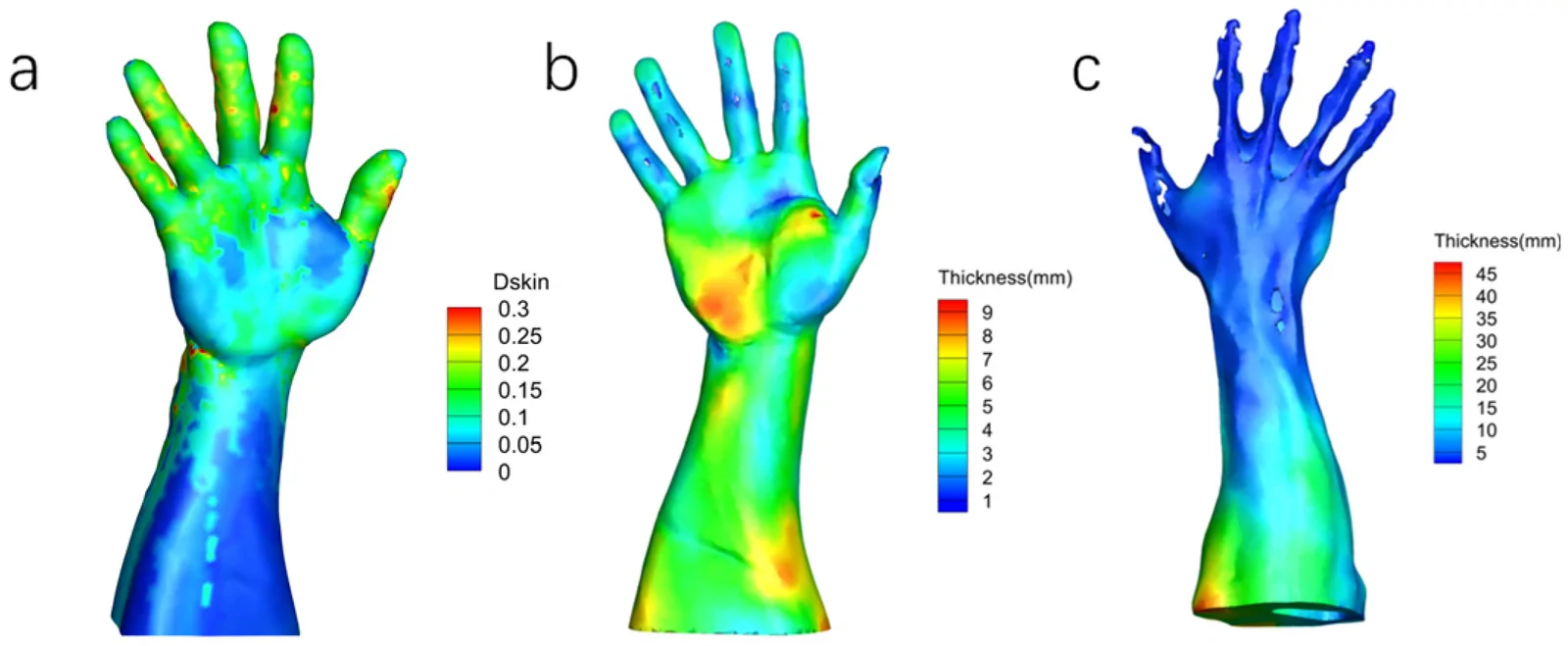
Spatial distributions of (**a**) the skin-dominance index, $D_{\mathrm{skin}}$; (**b**) fat thickness (mm); and (**c**) muscle thickness (mm) across the hand–forearm surface.

Analysis of the skin-dominance index ($D_{\mathrm{skin}}$) and the fat- and muscle-thickness fields demonstrated distinct spatial patterns across the hand–forearm surface (Figure 4).

P2 and P3 were selected to represent the maximum fat-thickness region and maximum muscle-thickness region, respectively.

For P1 selection, the skin-dominance index ($D_{\mathrm{skin}}$) was used to identify regions in which the skin layer contributed relatively strongly to the local soft-tissue thickness. As shown in Figure 4, relatively high $D_{\mathrm{skin}}$ values were observed on the fingers, particularly in the interdigital web spaces. However, the web spaces were unsuitable for indentation because their narrow and highly curved geometry, together with the proximity of adjacent digits, prevented stable and repeatable application of a cylindrical indenter.

**Figure 5:**
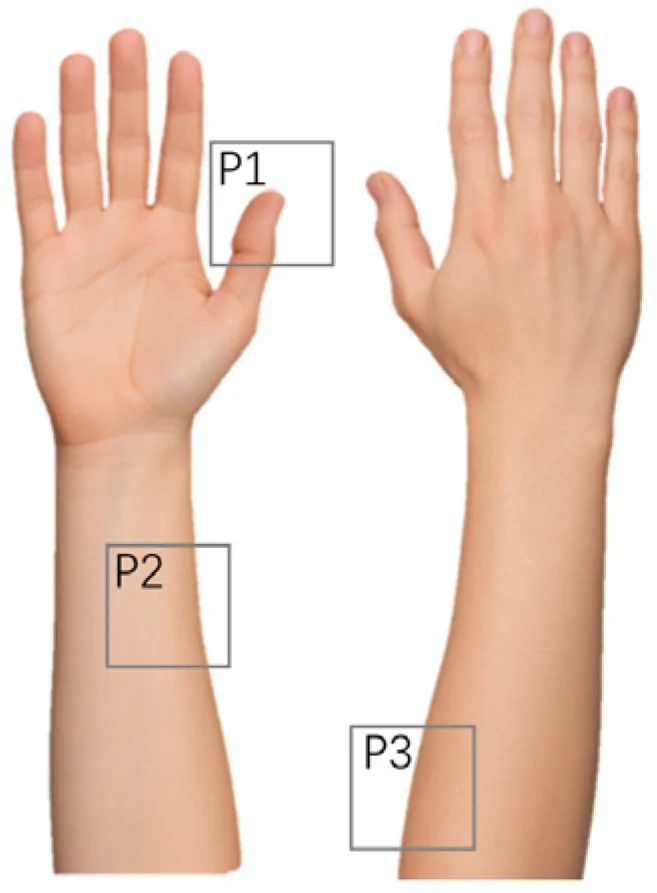
Selected indentation locations and local layer thicknesses based on the skin-dominance index ($D_{\mathrm{skin}}$) and the fat- and muscle-thickness fields.

Therefore, the thumb pulp, which exhibited a relatively high $D_{\mathrm{skin}}$ value while providing a broad and accessible surface for normal loading, was selected as P1 for the subsequent in vivo indentation experiment and finite element simulation. The final locations of P1–P3 and their corresponding local tissue measurements are shown in Figure 5.

### 3.4. Site-Specific Material-Combination Screening and Cross-Site Transfer Evaluation

Material-combination screening at P1 and P2

At P1, located over the distal thumb pulp, inspection of the reconstructed anatomy confirmed that no skeletal-muscle belly was present directly beneath the indentation region. This is consistent with the anatomical organization of the digital pulp. Therefore, only the nine skin–fat material combinations were considered at P1.

**Figure 6:**
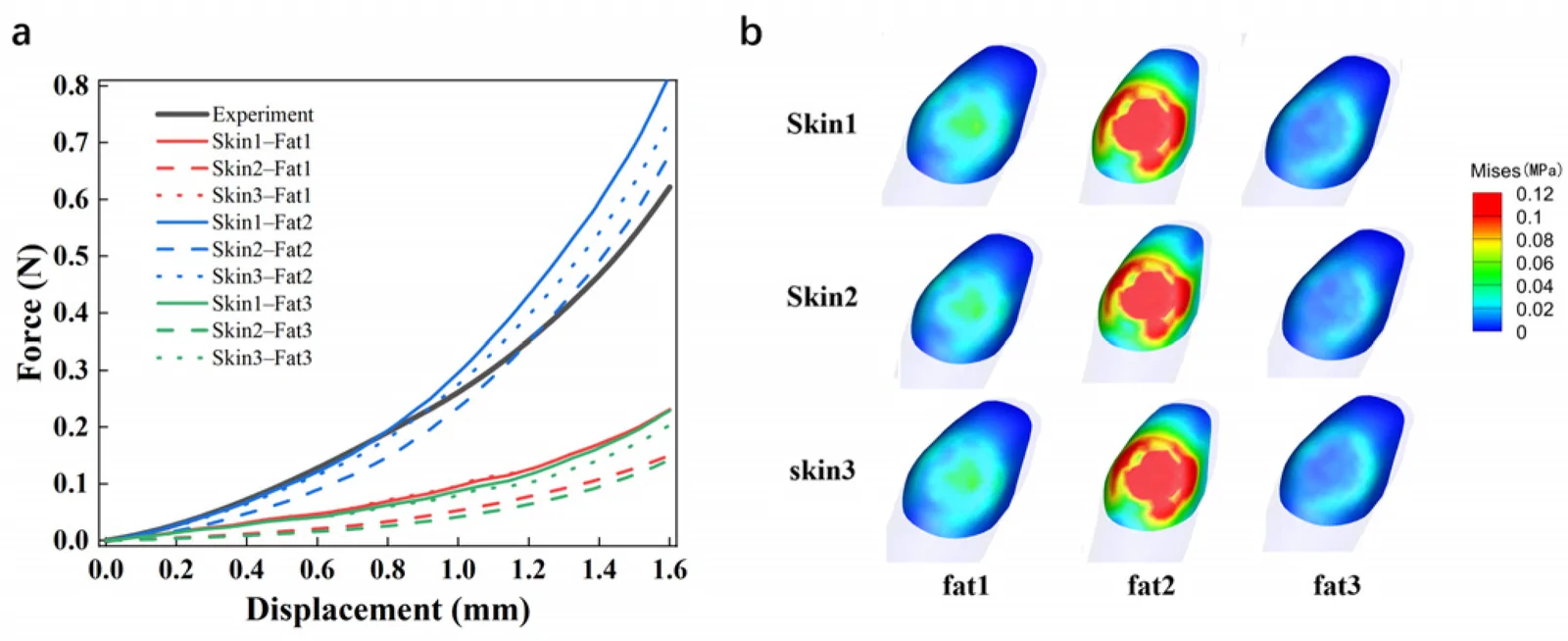
Force–displacement curves at P1 compared with the 9 skin–fat simulation combinations (**a**), together with the corresponding von Mises stress distributions (**b**).

At P1, the Fat2-containing combinations most closely tracked the experimental curve, whereas the Fat1- and Fat3-containing combinations remained below it (Figure 6). The Fat2-containing models also showed higher and more localized contact stresses than the Fat1- and Fat3-containing models.

Skin2–Fat2 achieved the best overall agreement at P1, with an RMSE of 0.029 N, an NRMSE of 0.047, an R^2^ of 0.972, and a peak-force error of 0.092. Skin3–Fat2 ranked second (NRMSE = 0.068; R^2^ = 0.942), followed by Skin1–Fat2 (NRMSE = 0.116; R^2^ = 0.829) (Table 7).

**Figure 7:**
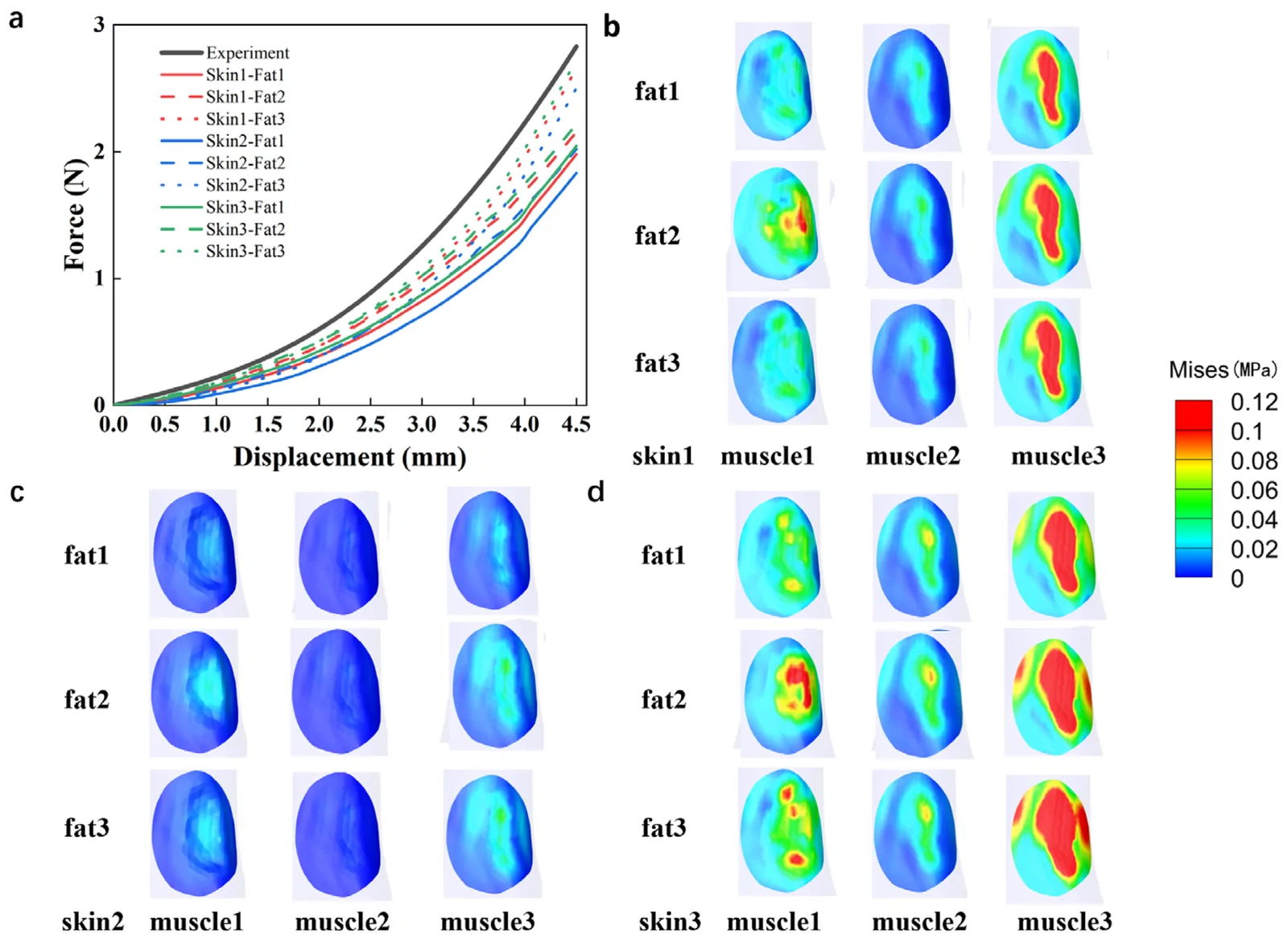
Force–displacement responses and corresponding contact-surface von Mises stress distributions at P2. (**a**) Experimental response and simulated responses for the 9 skin–fat pairings combined with Muscle3. (**b**–**d**) Contact-surface von Mises stress distributions for Skin1 (**b**), Skin2 (**c**), and Skin3 (**d**); rows indicate Fat1–Fat3 and columns indicate Muscle1–Muscle3.

At P2, Muscle1-containing curves consistently overestimated the experimental response, whereas Muscle2-containing curves underestimated it; Muscle3-containing curves were generally closest (Figure 7a). The stress maps showed predominantly low stresses for Muscle2, larger high-stress regions for Muscle3, and more heterogeneous local concentrations for Muscle1 (Figure 7b–d).

Skin3–Fat3–Muscle3 achieved the best overall agreement at P2, with an RMSE of 0.135 N, an NRMSE of 0.048, an R^2^ of 0.973, and a peak-force error of 0.037. Skin1–Fat3–Muscle3 ranked second (NRMSE = 0.063; R^2^ = 0.953), whereas Skin3–Fat2–Muscle3 ranked third (NRMSE = 0.093; R^2^ = 0.897) (Table 8).

Muscle3 provided the lowest NRMSE and highest $R^{2}$ for each matched skin–fat pairing at P2 and was therefore retained in the second stage of selection. Across the nine matched skin–fat pairs, Skin3–Fat2 produced the lowest mean NRMSE across P1 and P2 and the highest mean $R^{2}$. Skin3–Fat2–Muscle3 was therefore selected as the P1-P2 transfer candidate and was evaluated unchanged at P3.

Cross-site evaluation of the P1–P2 transfer candidate at P3

To assess cross-site transferability, the P1–P2 transfer candidate, Skin3–Fat2–Muscle3, was applied unchanged at P3. Compared with the experimental force–displacement response, this configuration produced an RMSE of 0.144 N, an NRMSE of 0.101, an R^2^ of 0.877, and a peak-force error of 0.192 (Table 9).

**Figure 8:**
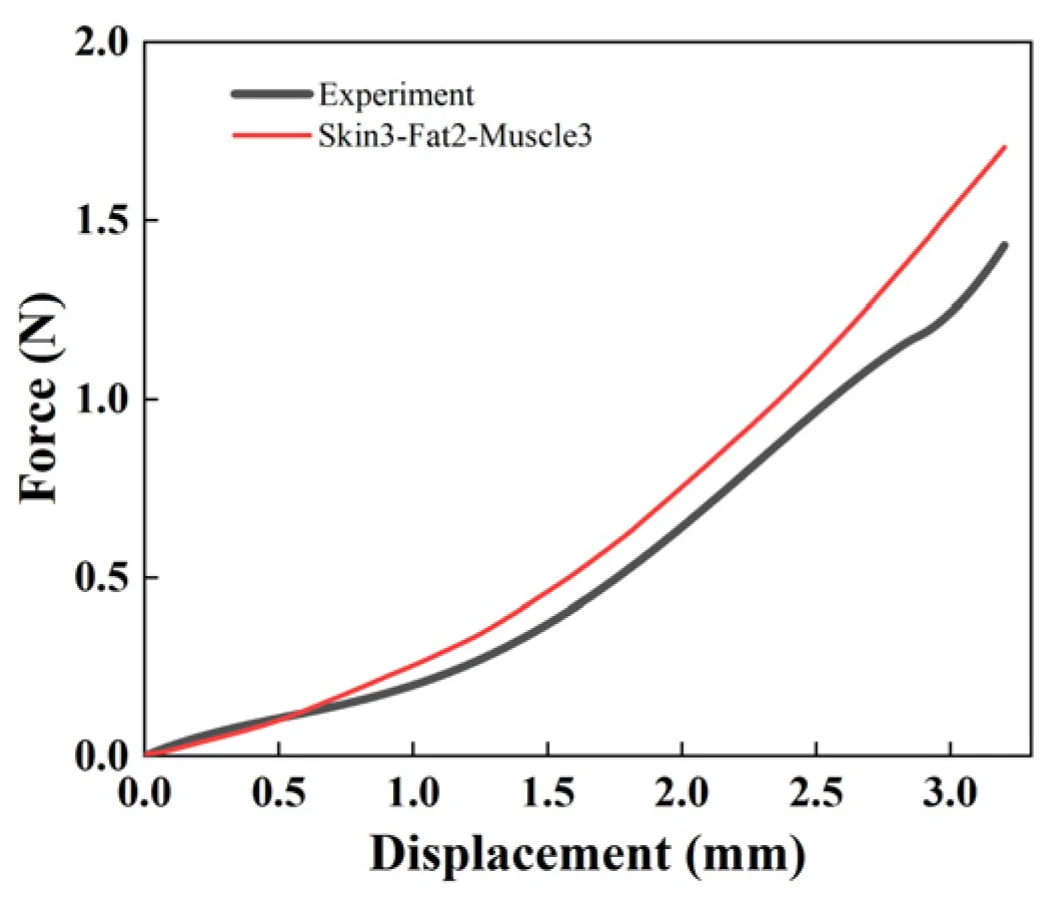
Comparison of the experimentally measured and model-predicted force–displacement responses for P3 Case Skin3–Fat2–Muscle3.

The prediction closely followed the experimental response at approximately 0–1.0 mm and reproduced the overall nonlinear stiffening trend, but it increasingly overestimated force at larger displacements (Figure 8). The corresponding subsurface maximum-principal logarithmic-strain field at a net indentation depth of 3.2 mm is shown in Supplementary Section S5.

Additional exploratory material-combination screening at P3

**Figure 9:**
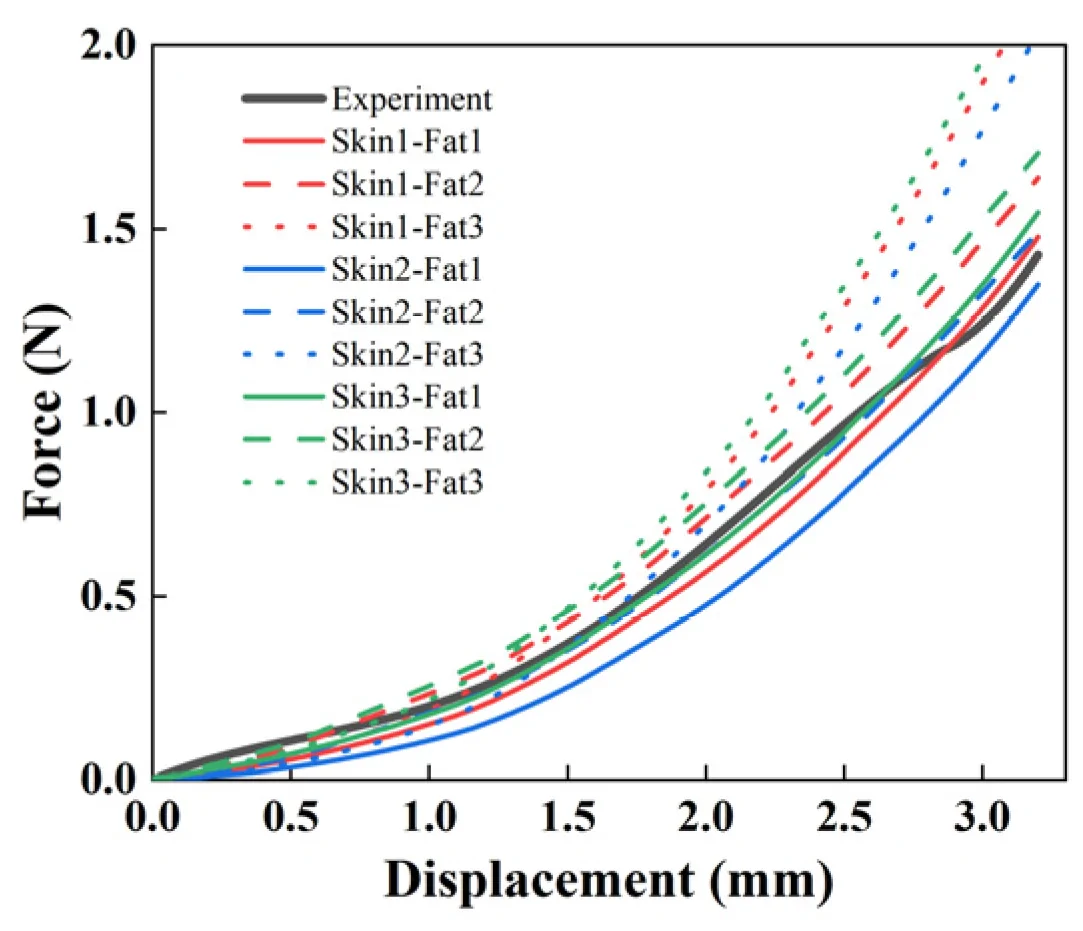
Exploratory force–displacement responses of the 9 skin–fat–muscle3 material combinations at P3 compared with the experimental curve.

After the cross-site evaluation, all 27 combinations were screened at P3 to identify the closest site-specific response and contextualize the transferred combination (Figure 9; Supplementary Section S4, Figure S4). No P3 result was used to select the transfer candidate.

The P3 responses were primarily stratified by muscle definition. Muscle1-containing combinations markedly overestimated the experimental terminal force, producing 3.17–10.03 N compared with the measured value of 1.43 N. Muscle2-containing combinations underestimated it, producing 0.24–0.62 N, whereas Muscle3-containing combinations were closest, producing 1.35–2.24 N.

Skin2–Fat2–Muscle3 provided the best overall curve agreement at P3, with an RMSE of 0.053 N, an NRMSE of 0.037, an R^2^ of 0.983, and a peak-force error of 0.044. Skin1–Fat1–Muscle3 showed the smallest peak-force error of 0.034. The transferred Skin3–Fat2–Muscle3 combination ranked sixth among the 27 candidates (NRMSE = 0.101; R^2^ = 0.877), whereas the P2-specific optimum, Skin3–Fat3–Muscle3, performed poorly at P3 (NRMSE = 0.232; R^2^ = 0.344) (Table 10).

## 4. Discussion

This study developed an MRI-based subject-specific finite element model of the hand and forearm that simultaneously represents bones, ligaments, muscle–tendon paths, skin, and subcutaneous fat, thereby providing an anatomically detailed platform for analyzing local surface loading. Layer-thickness mapping demonstrated that the hand–forearm surface should not be regarded as a homogeneous soft-tissue envelope, but rather as a spatially heterogeneous structure with distinct skin, fat, and muscle-thickness profiles. Representative indentation sites selected on the basis of these thickness differences exhibited different optimal material combinations. However, the results did not indicate universal non-transferability. The jointly selected P1–P2 candidate retained good agreement at P3 ($R^{2}$ = 0.877), whereas the P2-specific optimum decreased from $R^{2}$ = 0.973 at P2 to $R^{2}$ = 0.344 at P3. These findings indicate that cross-site transferability was material combination-dependent and that source-site optimality did not guarantee target-site optimality.

Compared with published hand, wrist, and forearm muscle parameters [14,38,39,40,41,42,43,44,45,46,47], most directly comparable muscle lengths fell within the reported literature ranges, suggesting that the MRI segmentation, geometric correction, and muscle–tendon boundary workflow preserved the principal scale of muscle-belly anatomy. Discrepancies were observed for AbPL and PI 2. These differences may reflect inter-specimen anatomical variability, differences in imaging posture and measurement protocols, and uncertainty in defining muscle–tendon boundaries during image-based reconstruction. In addition, the FPB and APB were represented as a combined model component, whereas the available literature reports these muscles separately. Therefore, the length comparison should be interpreted as a geometric consistency check rather than independent experimental validation. Future studies should evaluate the reconstructed geometry against subject-matched high-resolution imaging and anatomical measurements in a larger sample of participants.

Previous in vivo indentation studies have shown that surface stiffness varies substantially across anatomical sites in the extremities and that indentation responses are influenced by the composition and thickness of multilayer tissues, including skin, fat, and muscle [17,18]. By preserving continuous hand–forearm bony support, muscle–tendon paths, ligament constraints, and layered soft-tissue-thickness fields, the present model may support contact-pressure mapping, local force–displacement analysis, and assessment of soft-tissue deformation risk.

Cross-site results should distinguish site-specific optimality from predictive performance. Exploratory P3 screening identified Skin2–Fat2–Muscle3 as the P3-specific optimum (NRMSE = 0.037; R^2^ = 0.983); this candidate contained the P1-optimal Skin2–Fat2 pair but performed less well at P2 (NRMSE = 0.136; R^2^ = 0.780). Conversely, the jointly selected P1–P2 candidate was not P3-optimal but retained an NRMSE of 0.101 and an R^2^ of 0.877. Because P1 contained no muscle belly, P1-to-P2/P3 comparisons should be regarded as matched-pair exploratory analyses. Transferability must therefore be evaluated at the intended target site.

Previous studies similarly show that indentation-derived coefficients depend on participant, test site, loading rate, tissue thickness, indenter geometry, substrate curvature, and regional tissue mechanics [15,16,17,50,51,52,53]. Because the present study screened discrete literature-derived combinations rather than uniquely identifying tissue parameters, the selected combinations should be regarded as site- and loading-specific apparent representations rather than unique intrinsic tissue properties.

Local pressure risk should not be inferred solely from surface shape or total soft-tissue thickness, because the same external displacement may correspond to different combinations of skin, fat, muscle, tendon, and deep skeletal support. For indentation experiments and similar local loading tests, material parameters fitted at one region should not be directly extrapolated to other regions without validation. Evaluating multiple sites with distinct layer configurations may help identify region-specific apparent parameters and reduce overinterpretation based on single-point calibration.

There are several limitations to this study. First, owing to the considerable technical demands of constructing detailed image-based forearm models—including manual segmentation of closely packed muscles, complex contact definitions, and stringent mesh-quality requirements—the present study adopted a single-subject proof-of-concept design involving one healthy adult male. This restricts the generalizability of the findings to individuals of different ages, biological sexes, body sizes, tissue compositions, and pathological conditions. Second, the experimental assessment was restricted to three indentation locations. These locations were selected to represent markedly different local soft-tissue-thickness profiles, as identified from the mBRP maps of the hand and forearm before indentation testing. Nevertheless, the present study provides only a limited response-level cross-site assessment under the investigated passive indentation conditions and does not constitute comprehensive experimental validation of the finite element model. Future studies should include additional indentation locations and independent participants to evaluate spatial and inter-subject generalization.

## 5. Conclusions

This study developed an MRI-based subject-specific finite element model of the hand and forearm that incorporates bones, cartilage, ligaments, muscle–tendon paths, skin, and subcutaneous fat. The model was evaluated for anatomical consistency and local surface indentation responses at three sites with distinct layer-thickness configurations. Comparisons among literature-derived candidate skin–fat–muscle material combinations showed that local force–displacement behavior was influenced by layered-tissue thickness, deep anatomical support, and the combined response of multiple tissue layers. The material combination selected from P1 and P2 retained good agreement at the held-out P3 site, whereas the P2-specific optimum did not generalize uniformly to P3. The tested combinations should therefore be regarded as literature-derived modeling choices whose suitability requires evaluation for the intended site and loading condition, rather than as universally applicable intrinsic tissue properties. The findings are limited to one healthy adult male and the investigated passive indentation conditions; multi-participant studies are required before population-level or application-specific generalization.

## Figures and Tables

**Table 1:** Anatomical origins and insertions of modeled muscle–tendon units.

Region	Muscle	Abbreviation	Origin	Insertion
Forearm	Flexor carpi radialis	FCR	Hum	MC2
Forearm	Flexor carpi ulnaris	FCU	Hum/Uln	MC3, MC5
Forearm	Flexor digitorum superficialis	FDS	Hum/Uln	MP2, MP3, MP4, MP5
Forearm	Flexor digitorum profundus	FDP	Uln/Rad	DP2, DP3, DP4, DP5
Forearm	Flexor pollicis longus	FPL	Rad	DP1
Forearm	Extensor carpi radialis longus	ECRL	Hum/Rad	MC2
Forearm	Extensor carpi radialis brevis	ECRB	Hum/Rad	MC3
Forearm	Extensor carpi ulnaris	ECU	Hum/Uln	MC5
Forearm	Extensor digitorum communis	ED	Hum	DP2, DP3, DP4, DP5
Forearm	Extensor digiti minimi	EDM	Hum	DP5
Forearm	Abductor pollicis longus	AbPL	Rad/Uln	MC1
Forearm	Pronator quadratus	PQ	Uln	Rad
Hand	Opponens digiti minimi	ODM	Carpal bones	MC5
Hand	Abductor digiti minimi	ADM	Carpal bones	PP5
Hand	Flexor digiti minimi	FDM	Carpal bones	PP5
Hand	Opponens pollicis	OpP	Carpal bones	MC1
Hand	Adductor pollicis	AdP	MC2/MC3	PP1
Hand	Flexor pollicis brevis + abductor pollicis brevis	FPB + APB	Carpal bones	PP1
Hand	First lumbrical	Lum 1	DP2/MP2	Extensor expansion
Hand	Second lumbrical	Lum 2	DP2/MP3	Extensor expansion
Hand	Third lumbrical	Lum 3	DP2/MP4	Extensor expansion
Hand	Fourth lumbrical	Lum 4	DP2/MP5	Extensor expansion
Hand	First dorsal interosseous	DI 1	MC2	PP2
Hand	Second dorsal interosseous	DI 2	MC2/MC3	PP3
Hand	Third dorsal interosseous	DI 3	MC3/MC4	PP3
Hand	Fourth dorsal interosseous	DI 4	MC4/MC5	PP4
Hand	First palmar interosseous	PI 1	MC2/MC3	PP2
Hand	Second palmar interosseous	PI 2	MC4	PP4
Hand	Third palmar interosseous	PI 3	MC5	PP5

Abbreviations (origins and insertions): Hum, humerus; Rad, radius; Uln, ulna; MC1–MC5, first to fifth metacarpals; PP1–PP5, proximal phalanges; MP2–MP5, middle phalanges; DP1–DP5, distal phalanges of the respective digits.

**Table 2:** Representative literature-derived regional full-thickness skin values prescribed for model construction.

Skin Area	Volar Forearm	Dorsal Forearm	Wrist	Dorsum of Hand	Palm	Finger
Thick (mm)	1.13	1.34	1.36	1.20	1.85	1.20
Ref.	[24]	[24]	[25]	[25]	[26]	[25]

**Table 3:** Numerical verification of the mBRP thickness-recovery procedure using prescribed regional skin thicknesses.

Region	Ref (mm)	mBRP (mm)	Err (%)
Finger	1.20	1.22	1.67
Palm	1.85	1.87	1.08
Dorsum of hand	1.20	1.21	0.83
Wrist	1.36	1.37	0.74
Dorsal forearm	1.34	1.34	0.00
Volar forearm	1.13	1.13	0.00

**Table 4:** Candidate hyperelastic constitutive models and associated parameters for skin, fat, and muscle.

Tissue	Model	Formulation	Parameter	Ref.
Skin	N-H	$W=C_{10}\left({\bar{I}}_{1}-3\right)+\frac{K}{2}(J-1{)}^{2}$	$C_{10}=120$ K=5450	[31]
	M-R + P(2)	$W=C_{10}\left({\overset{‾}{I}}_{1}-3\right)+C_{11}\left({\overset{‾}{I}}_{1}-3\right)\left({\overset{‾}{I}}_{2}-3\right)+\frac{1}{D_{1}}(J-1{)}^{2}$ $g(t)=1-\sum_{i=1}^{2}g_{i}\left(1-e^{-\frac{t}{\tau_{i}}}\right)$	$C_{10}=9.4$ $C_{11}=82$ $D_{1}=0.001$ $g_{i}=\{0.30,0.35\}$ $\tau_{1}=\{0.37,5.59\}$	[32]
	O(1)	$W=\frac{\mu_{1}}{\alpha_{1}}\left({\bar{\lambda }}_{1}^{\alpha_{1}}+{\bar{\lambda }}_{2}^{\alpha_{1}}+{\bar{\lambda }}_{3}^{\alpha_{1}}-3\right)+\frac{1}{D_{1}}(J-1{)}^{2}$	$\mu_{1}=56.8$ $\alpha_{1}=13.3$ $D_{1}=7.45\times 10^{-5}$	[33]
Fat	N-H + P(1)	$W=\mu /2\left({\bar{I}}_{1}-3\right)$ $g(t)=1-g_{1}\left(1-e^{-\frac{t}{\tau_{1}}}\right)$	μ=0.53 $g_{1}=0.39$ τ_1_ = 700	[34]
	O(4)	$W=\sum_{p=1}^{N}\frac{C_{p}\left(\lambda_{1}^{2m_{p}}+\lambda_{2}^{2m_{p}}+\lambda_{3}^{2m_{p}}-3\right)}{2m_{p}}$	$C_{i}=\{2.34,4.08,-0.42,1.34\}$ $m_{i}=\{1,-1,2,-2\}$	[35]
	O(3)	$W=\sum_{p=1}^{n}\;\frac{\mu_{p}}{\alpha_{p}}\left(\lambda_{1}^{\alpha_{p}}+\lambda_{2}^{\alpha_{p}}+\lambda_{3}^{\alpha_{p}}-3\right)$	$\mu_{i}=\{49,9,40\}$ $\alpha_{i}=\{5.51,6.75,5.26\}$	[9]
Muscle	O(1) + P(5)	$W=\frac{\mu }{\alpha }\left({\bar{\lambda }}_{1}^{\alpha }+{\bar{\lambda }}_{2}^{\alpha }+{\bar{\lambda }}_{3}^{\alpha }-3\right)+\frac{1}{D}(J-1{)}^{2}$ $G(t)=\sum_{i=1}^{n}\;G_{i}e^{-\beta_{i}t}$	$\mu_{1}=13.37$ α=14.5 $D=3.02\times 10^{-3}$ $G_{i}=\{522,211,375,290,80\}$ $\beta_{i}=\{1.01,0.4,0.07,0.03,0.0001\}$	[36]
	O(1)	$W=\frac{2\mu }{\alpha^{2}}\left({\bar{\lambda_{1}}}^{\alpha }+{\bar{\lambda_{2}}}^{\alpha }+{\bar{\lambda_{3}}}^{\alpha }-3\right)+\frac{1}{D}\left(J^{el}-1\right)$	μ=1.675 α=22 D=0.024	[37]
	Po(2) + P(3)	$W=C_{10}\left({\bar{I}}_{1}-3\right)+C_{01}\left({\bar{I}}_{2}-3\right)+\frac{1}{D_{1}}(J-1{)}^{2}$ $g(t)=1-\sum_{i=1}^{3}g_{i}\left(1-e^{-\frac{t}{\tau_{i}}}\right)$	$C_{10}=0.6$ $C_{01}=0.55$ $D_{1}=0.0175$ $g_{i}=\{0.89,0.05,0.03\}$ τ={0.362,2.54,52.0}	[38]

Note: N–H, neo-Hookean; M–R, Mooney–Rivlin; O(n), n-term Ogden; Po(n), nth-order polynomial; and P(n), n-term Prony series. The plus sign (+) indicates a combination of hyperelastic and viscoelastic formulations. Units are kPa for C, K, μ, and G; ${kPa}^{-1}$ for D; s for τ; and ${ms}^{-1}$ for β. The parameters α, m, and g are dimensionless. The Prony-series parameters are reported as part of the source formulations but were not active in the present Static, General analyses.

**Table 5:** Anthropometric comparison with GB/T 10000-2023 male P50 reference values.

Measure	P50 (mm)	This Study (mm)	Deviation (%)
Elbow–wrist length	235	241.05	+2.57
Hand length	184	184.6	+0.33
Hand breadth	88	89.62	+1.84
Index finger length	72	72.76	+1.06
Index finger proximal breadth	20	20.05	+0.25

**Table 6:** Comparison of model-derived muscle-belly lengths with published anatomical measurements. The reference value or range and corresponding literature source for each muscle are provided in the table.

Muscle	Model Belly Length (mm)	Reference Belly Length (mm)	Ref.
FCR	164.18	137.6–185.4	[40]
FCU	271.94	243–325	[41]
FDS	182.53	143.7–187.9	[14,40]
FDP	132.08	110.3–147.7	[14,40]
FPL	64.46	64	[14,40,41,42,43,44,45,46,47,48,49]
ECRL	182.16	124.4–186.2	[49]
ECRB	195.38	166.3–206.5	[49]
ECU	246.51	200.4–256.0	[40,41,42]
ED	274.36	235–279	[42,49]
EDM	225.72	142.8–251	[42,49]
AbPL	126.87	134–176	[47]
PQ	55.95	46.6–66.6	[46]
ODM	44.64	43.6–50.8	[47]
ADM	68.15	61.9–74.9	[47]
FDM	60.78	48.8–69.6	[47]
OpP	45.54	45.5–65.5	[47]
AdP	53.70	45.7–63.5	[47]
FPB + APB	59.56	FPB 53.5–60.9 APB 53.8–67.0	[47]
Lum 1	59.52	53–86	[45]
Lum 2	81.88	52–82	[45]
Lum 3	51.23	38.7–63.7	[44,45]
Lum 4	49.14	46–78	[45]
DI 1	52.62	45–80	[43]
DI 2	57.18	54.7–70.9	[47]
DI 3	44.97	44.81–53.23	[43,47]
DI 4	51.46	44.8–55.4	[47]
PI 1	45.49	45.1–65.1	[47]
PI 2	55.35	45.3–51.1	[47]
PI 3	41.73	39.5–51.1	[47]

**Table 7:** Quantitative agreement between the experimental and simulated force–displacement curves for the nine skin–fat material combinations at P1.

Combination	RMSE (N)	NRMSE	R^2^	Peak-Force Error
Skin1–Fat1	0.181	0.291	−0.073	0.629
Skin1–Fat2	0.072 (3)	0.116 (3)	0.829 (3)	0.318 (3)
Skin1–Fat3	0.186	0.299	−0.136	0.631
Skin2–Fat1	0.221	0.356	−0.610	0.759
Skin2–Fat2	0.029 (1)	0.047 (1)	0.972 (1)	0.092 (1)
Skin2–Fat3	0.230	0.370	−0.734	0.772
Skin3–Fat1	0.180	0.290	−0.068	0.630
Skin3–Fat2	0.042 (2)	0.068 (2)	0.942 (2)	0.189 (2)
Skin3–Fat3	0.197	0.316	−0.269	0.673

Note: The experimental peak force at P1 was 0.62 ± 0.03 N, reported as the mean ± SD across three repeated measurements. Numerals (1), (2), and (3) indicate the first-, second-, and third-ranked values within each evaluation-metric column, respectively.

**Table 8:** Quantitative agreement between the experimental and simulated force–displacement curves for the 27 skin–fat–muscle material combinations at P2.

Combination	RMSE (N)	NRMSE	R^2^	Peak-Force Error
Skin1–Fat1–Muscle1	3.214	1.136	−14.436	2.543
Skin1–Fat1–Muscle2	1.024	0.362	−0.566	0.822
Skin1–Fat1–Muscle3	0.425	0.150	0.731	0.300
Skin1–Fat2–Muscle1	13.987	4.943	−291.281	13.915
Skin1–Fat2–Muscle2	0.990	0.350	−0.464	0.804
Skin1–Fat2–Muscle3	0.300	0.106	0.866	0.249
Skin1–Fat3–Muscle1	7.974	2.818	−93.979	7.362
Skin1–Fat3–Muscle2	1.005	0.355	−0.510	0.808
Skin1–Fat3–Muscle3	0.178 (2)	0.063 (2)	0.953 (2)	0.061 (2)
Skin2–Fat1–Muscle1	2.369	0.837	−7.383	1.920
Skin2–Fat1–Muscle2	1.128	0.399	−0.900	0.883
Skin2–Fat1–Muscle3	0.518	0.183	0.600	0.352
Skin2–Fat2–Muscle1	7.782	2.750	−89.480	6.912
Skin2–Fat2–Muscle2	1.084	0.383	−0.755	0.859
Skin2–Fat2–Muscle3	0.383	0.136	0.780	0.286
Skin2–Fat3–Muscle1	7.207	2.547	−76.603	6.879
Skin2–Fat3–Muscle2	1.115	0.390	−0.856	0.874
Skin2–Fat3–Muscle3	0.276	0.098	0.886	0.116 (3)
Skin3–Fat1–Muscle1	3.360	1.187	−15.868	2.649
Skin3–Fat1–Muscle2	0.981	0.347	−0.438	0.800
Skin3–Fat1–Muscle3	0.384	0.136	0.779	0.277
Skin3–Fat2–Muscle1	11.635	4.111	−201.221	9.932
Skin3–Fat2–Muscle2	0.950	0.336	−0.347	0.781
Skin3–Fat2–Muscle3	0.263 (3)	0.093 (3)	0.897 (3)	0.216
Skin3–Fat3–Muscle1	8.117	2.868	−97.421	7.457
Skin3–Fat3–Muscle2	0.961	0.339	−0.378	0.781
Skin3–Fat3–Muscle3	0.135 (1)	0.048 (1)	0.973 (1)	0.037 (1)

Note: The experimental peak force at P2 was 2.83 ± 0.12 N, reported as the mean ± SD across three repeated measurements. Numerals (1), (2), and (3) indicate the first-, second-, and third-ranked values within each evaluation-metric column, respectively.

**Table 9:** Quantitative cross-site evaluation of the selected Skin3–Fat2–Muscle3 material combination at P3.

Combination	RMSE (N)	NRMSE	R^2^	Peak-Force Error
Skin3–Fat2–Muscle3	0.144	0.101	0.877	0.192

Note: The experimental peak force at P3 was 1.43 ± 0.06 N, reported as the mean ± SD across three repeated measurements.

**Table 10:** Quantitative agreement between the experimental and simulated force–displacement curves for the 27 skin–fat–muscle combinations in the exploratory post hoc analysis at P3.

Combination	RMSE (N)	NRMSE	R^2^	Peak-Force Error
Skin1–Fat1–Muscle1	0.913	0.638	−3.969	1.622
Skin1–Fat1–Muscle2	0.479	0.335	−0.367	0.735
Skin1–Fat1–Muscle3	0.066 (3)	0.046 (3)	0.974 (3)	0.034 (1)
Skin1–Fat2–Muscle1	2.915	2.038	−49.625	5.202
Skin1–Fat2–Muscle2	0.415	0.290	−0.024	0.616
Skin1–Fat2–Muscle3	0.109	0.076	0.929	0.147
Skin1–Fat3–Muscle1	2.715	1.899	−42.919	5.537
Skin1–Fat3–Muscle2	0.435	0.304	−0.128	0.656
Skin1–Fat3–Muscle3	0.296	0.207	0.479	0.519
Skin2–Fat1–Muscle1	0.643	0.450	−1.461	1.214
Skin2–Fat1–Muscle2	0.562	0.393	−0.885	0.835
Skin2–Fat1–Muscle3	0.123	0.086	0.911	0.057 (3)
Skin2–Fat2–Muscle1	2.588	1.810	−38.897	6.015
Skin2–Fat2–Muscle2	0.482	0.337	−0.385	0.720
Skin2–Fat2–Muscle3	0.053 (1)	0.037 (1)	0.983 (1)	0.044 (2)
Skin2–Fat3–Muscle1	2.379	1.664	−32.722	5.022
Skin2–Fat3–Muscle2	0.521	0.364	−0.618	0.761
Skin2–Fat3–Muscle3	0.231	0.162	0.683	0.425
Skin3–Fat1–Muscle1	1.001	0.700	−4.968	1.740
Skin3–Fat1–Muscle2	0.438	0.306	−0.142	0.686
Skin3–Fat1–Muscle3	0.061 (2)	0.043 (2)	0.978 (2)	0.081
Skin3–Fat2–Muscle1	3.114	2.178	−56.762	5.520
Skin3–Fat2–Muscle2	0.382	0.267	0.130	0.568
Skin3–Fat2–Muscle3	0.144	0.101	0.877	0.192
Skin3–Fat3–Muscle1	2.818	1.971	−46.302	5.691
Skin3–Fat3–Muscle2	0.393	0.275	0.080	0.606
Skin3–Fat3–Muscle3	0.332	0.232	0.344	0.567

Note: The experimental peak force at P3 was 1.43 ± 0.06 N, reported as the mean ± SD across three repeated measurements. (1), (2), and (3) indicate the first-, second-, and third-ranked values within each evaluation-metric column, respectively.

## Data Availability

The processed data and analysis materials supporting the findings of this study are available from the corresponding author upon reasonable request.

## Supplementary Materials

The following supporting information can be downloaded at https://www.mdpi.com/article/10.3390/bioengineering13091015/s1: Section S1, Comparison with representative finite element models [7,8,9,14,16,19,20]; Section S2, Modified bidirectional ray-projection and multi-method thickness-estimation workflow; Section S3, Mesh convergence analysis; Section S4, Additional force–displacement responses separated by muscle material set; Section S5, Sub-surface strain distribution at P3.
