# Supplementary material for: Development and Surface Indentation Assessment of an MRI-Based Subject-Specific Hand–Forearm Finite Element Model: A Preliminary Study
Source: Bioengineering (Basel). 2026 Aug 31;13(9):1015. doi: 10.3390/bioengineering13091015 (PMC13603692; doi:10.3390/bioengineering13091015)
Supplement: Supplementary file 1 [file bioengineering-13-01015-s001.zip › bioengineering-4446932-supplementary.pdf]

Supplementary Materials

Section S1 Comparison with representative finite element models

To clarify the anatomical contribution of the present model, representative hand, forearm, fingertip, and indentation finite element models were compared in terms of anatomical extent, tissue representation, muscle–tendon continuity, subject-specificity, experimental evaluation, and numbers of subjects and evaluation sites (Table S1).

Table S1. Comparison of representative finite element models relevant to the present study.

Ref.	Extent	Tissues	MT continuity	Basis/sample	Evaluation
[20]	3D fingertip	Skin/subcut./bone/nail	No	Generic; pub. data	Static/dynamic FD
[7]	2D fingertip	Layered skin/subcut.; ridges	No	Generic	Static/sliding contact
[8]	3D hand/wrist	Bone/ST envelope	No	CT-based; n=1 test	CA during cylindrical grasp
[9]	3D hand/wrist	Bone/lig./tendon/skin/subcut.	Tendon paths; no forearm MB	SS; n=1; 3 tasks	In-vivo CP and CA
[14]	3D forearm/wrist	Bone/muscle/tendon/lig./cart.	Partial; forearm only	SP; n=1	Geometry, mesh, axial load
[16]	Local forearm	Skin/fat/muscle	No	Image-based; n=11	Indentation FD matching
[19]	3D transtibial residuum	Skin–fat/muscle–ST	N/A	SS; n=1; 18 sites	Inverse FE: 4 cal./14 pred.
Our study	3D hand–forearm–humerus	Bone/cart./lig./tendon/muscle/skin/fat	Yes	SS; n=1; 3 sites	In-vivo FD and cross-site test

FE, finite element; MT, muscle–tendon; MB, muscle belly; ST, soft tissue; SS, subject-specific; SP, specimen-specific; FD, force–displacement; CP, contact pressure; CA, contact area; subcut., subcutaneous tissue; lig., ligament; cart., cartilage; pub., published; cal., calibration; pred., prediction; N/A, not applicable.

Section S2 Modified bidirectional ray-projection and multi-method thickness-estimation workflow

The soft-tissue models were represented by triangulated surface meshes. For a closed tissue mesh containing both the inner and outer boundaries, the two surfaces could not be reliably separated using the original surface orientation alone because of local concavities, finger gaps, open boundaries, and inconsistently oriented surface normals. A modified bidirectional ray-projection (mBRP) procedure incorporating three consecutive screening steps was therefore implemented.

Section S2.1 Consecutive screening

<u>Section S2.1.1 Local normal-consistency screening</u>

For each surface vertex p_i, the consistency of its unit normal vector n_i with those of the neighbouring vertices located within a radius of 3 mm was evaluated using the mean dot product:

$$C_i = \frac{1}{N_i} \sum_{j \in \mathcal{N}_i} n_i \cdot n_j$$

where $\mathcal{N}_i$ denotes the set of neighbouring vertices within a radius of 3 mm and N_i is the number of vertices in this neighbourhood. Vertices with $C_i<0.5$ were considered to lie in geometrically unstable regions, such as finger-gap boundaries, model openings, or sharply changing surface regions, and were excluded from subsequent projection.

Section S2.1.2 Bidirectional ray-pair validation

From each retained vertex, rays were cast along both the positive and negative directions of its local normal:

$$r_i^{\pm}(t) = p_i \pm t n_i, \quad t > 0$$

Intersections between these rays and the tissue surface were identified. A candidate intersection was retained only when the normal vector of the intersected triangle was approximately opposite to the normal at the ray origin. Specifically, a pair was considered valid when

$$n_i \cdot n_{int} < -0.3$$

This condition reduced erroneous pairing with adjacent regions or with triangles located on the same side of the tissue layer.

Section S2.1.3 Anatomy-based inner–outer classification

For each valid point pair, the minimum distance from each point to the underlying bone surfaces was calculated. The point located closer to the bones was classified as belonging to the inner surface, whereas the point located farther from the bones was classified as belonging to the outer surface. This anatomy-based criterion allowed the inner and outer boundaries to be identified without relying solely on the original orientation of the STL surface normals.

Section S2.2 Local thickness estimation and quality control

Following surface pairing, three local thickness estimates were calculated. The first was the distance obtained from the bidirectional normal-ray projection. The second was calculated by projecting a ray from the outer-surface point towards its nearest point on the bone surface and identifying the first valid intersection with the inner tissue boundary. The third was the nearest Euclidean distance from the outer-surface point to the set of inner-surface points, calculated using a KD-tree spatial index.

The smallest valid value among the three estimates was initially selected because an oblique ray through the tissue layer produces a longer path than the corresponding locally perpendicular distance. An additional quality-control rule was applied to prevent the nearest-surface method from incorrectly matching a point on the same surface near an opening or boundary. When the nearest-surface distance was smaller than one-third of the smaller ray-based estimate, it was considered unreliable and was excluded. The smaller of the two ray-based estimates was then retained. Points for which no sufficiently reliable thickness estimate could be established were excluded from the final thickness field.

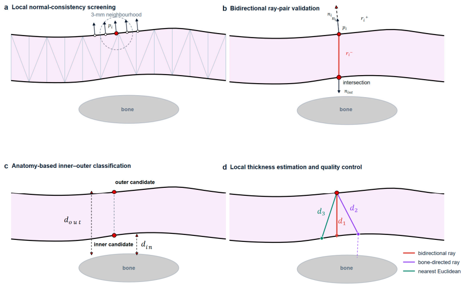

Figure S1. Schematic illustration of the modified bidirectional ray-projection (mBRP) procedure for inner–outer surface classification and local tissue-thickness estimation. (a) For each candidate vertex p_i, the consistency of its local normal vector n_i with those of neighbouring vertices within a 3-mm radius was evaluated; vertices with $C_i < 0.5$ were excluded. (b) Rays r_i^+ and r_i^- were projected along both directions of n_i. An intersection pair was accepted when the normal of the intersected facet, n_{int}, satisfied n_i*n_{int}<-0.3. (c) For each valid pair, the point closer to the underlying bone was classified as the inner-surface point, whereas the farther point was classified as the outer-surface point. (d) Three candidate thicknesses were calculated: the bidirectional normal-ray distance d_1, the bone-directed ray distance d_2, and the nearest Euclidean distance d_3. The smallest valid estimate was retained. When d_3 was less than one-third of the smaller ray-based estimate, it was considered unreliable and excluded.

Section S3 Mesh convergence analysis

A local mesh-convergence assessment was conducted by progressively refining the meshes at P1, P2, and P3, while keeping the mesh in all other regions unchanged. At each site, the baseline local mesh (M1) was compared with an intermediate mesh (M2) and the finest mesh (M3)(Table S2). Using M3 as the reference, the whole-curve NRMSE values for M1/M2 were 1.58%/0.78%, 0.73%/1.04%, and 0.54%/0.21% at P1, P2, and P3, respectively. The corresponding terminal-force differences were 4.25%/1.94%, 0.62%/2.17%, and 0.59%/0.49%, respectively. Although a small non-monotonic variation occurred at P2, all differences remained below 5%, and the force–displacement curves were closely aligned (Figure S2). Therefore, M1 was considered sufficiently mesh-independent for the global indentation response and was retained for the subsequent simulations.

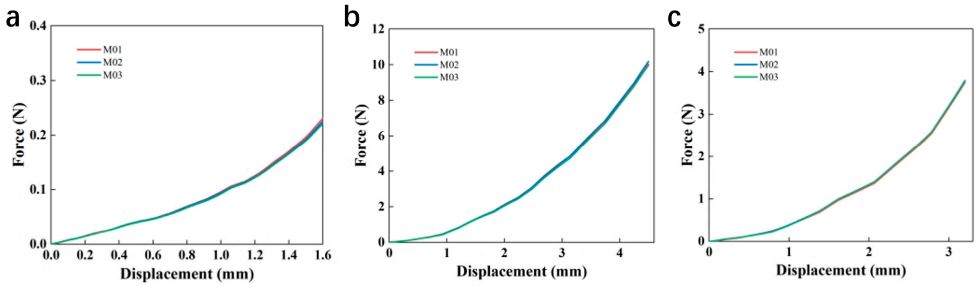

Figure S2. Force–displacement responses obtained using the M1, M2, and M3 meshes at (a) P1, (b) P2, and (c) P3.

Table S2. Characteristics of the locally refined meshes and convergence metrics relative to M3 at P1, P2, and P3.

Site	Local element count, M1/M2/M3	Whole-curve NRMSE relative to M3, M1/M2 (%)	Terminal-force difference relative to M3, M1/M2 (%)
P1	1,356 / 3,388 / 8,776	1.58 / 0.78	4.25 / 1.94
P2	2,262 / 5,731 / 15,038	0.73 / 1.04	0.62 / 2.17
P3	6,193 / 15,887 / 41,324	0.54 / 0.21	0.59 / 0.49

Section S4 Additional force – displacement responses separated by muscle material set

To improve the readability of the multi-curve plots, the force–displacement responses were separated according to the muscle material set. The Muscle3-containing combinations, which showed the closest overall agreement with the experimental responses, are presented in Figures 7a and 9 of the main manuscript. The corresponding Muscle1- and Muscle2-containing combinations at P2 and P3 are presented separately in

Figures S3 and S4. Together, the main and supplementary figures present the force–displacement responses of muscle1 and muscle2 material combinations at each location. Because the predicted force magnitudes differed substantially among the muscle material sets, separate force-axis ranges were used to maintain curve readability.

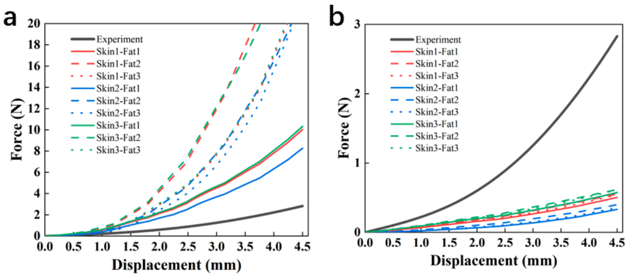

Figure S3. Force–displacement responses at P2 for the material combinations containing (a) Muscle1 and (b) Muscle2.

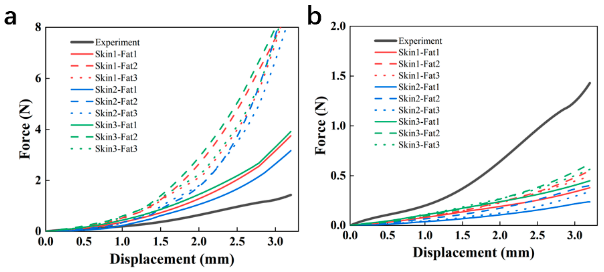

Figure S4. Force–displacement responses at P3 for the material combinations containing (a) Muscle1 and (b) Muscle2.

Section S5 Sub-surface strain distribution at P3

To visualize the subsurface deformation predicted by the layered finite element model, a longitudinal section through the P3 indentation region was examined for the Skin3–Fat2–Muscle3 material combination at a net indentation depth of 3.2 mm. The maximum principal logarithmic strain was concentrated immediately beneath the indenter and gradually decreased with increasing depth and lateral distance from the indentation region (Figure S5). This distribution illustrates the model-predicted spatial distribution of sub-surface deformation and is not interpreted as independent validation because no internal tissue-strain measurements were available for comparison. For visualization, the surrounding tissue geometry was rendered translucently to provide anatomical context.

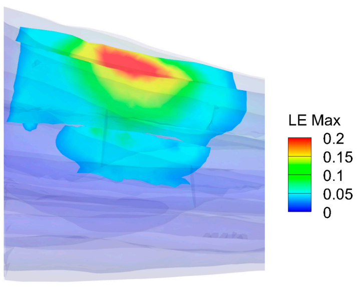

Figure S5. Cross-sectional distribution of the maximum principal logarithmic strain (LE_{max}, dimensionless) beneath the indenter at P3 for the specified Skin3–Fat2–Muscle3 combination at a net indentation depth of 3.2 mm.
